# Reducing and deducing the structures of consciousness through meditation

**DOI:** 10.3389/fpsyg.2022.884512

**Published:** 2022-09-08

**Authors:** Sucharit Katyal

**Affiliations:** Max Planck UCL Centre for Computational Psychiatry and Ageing Research, University College London, London, United Kingdom

**Keywords:** meditation, consciousness, phenomenology, Tantra Yoga, Ananda Marga, states of consciousness, transcendent, non-dual

## Abstract

According to many first-person accounts, consciousness comprises a subject-object structure involving a mental action or attitude starting from the “subjective pole” upon an object of experience. In recent years, many paradigms have been developed to manipulate and empirically investigate the object of consciousness. However, well-controlled investigation of subjective aspects of consciousness has been more challenging. One way, subjective aspects of consciousness are proposed to be studied is using meditation states that alter its subject-object structure. Most work to study consciousness in this way has been done using Buddhist meditation traditions and techniques. There is another meditation tradition that has been around for at least as long as early Buddhist traditions (if not longer) with the central goal of developing a fine-grained first-person understanding of consciousness and its constituents by its manipulation through meditation, namely the Tantric tradition of Yoga. However, due to the heavy reliance of Yogic traditions on the ancient Indian Samkhya philosophical system, their insights about consciousness have been more challenging to translate into contemporary research. Where such translation has been attempted, they have lacked accompanying phenomenological description of the procedures undertaken for making the precise subject-object manipulations as postulated. In this paper, I address these issues by first detailing how Tantric Yoga philosophy can be effectively translated as a systematic phenomenological account of consciousness spanning the entirety of the subject-object space divided into four “structures of consciousness” from subject to object. This follows from the work of the 20th century polymath and founder of the Tantric Yoga school of Ananda Marga, Prabhat Ranjan Sarkar, who expounded on the “cognitivization” of Samkhya philosophy. I then detail stepwise meditation procedures that make theoretical knowledge of these structures of consciousness a practical reality to a Tantric Yoga meditator in the first-person. This is achieved by entering meditative states through stepwise experiential reduction of the structures of consciousness from object to subject, as part of their meditative goal of “self-realization.” I end by briefly discussing the overlap of these putative meditation states with proposed states from other meditation traditions, and how these states could help advance an empirical study of consciousness.

## Introduction

While the scientific investigation of consciousness is a nascent field, a few decades old (Crick and Koch, 1992), the investigation of consciousness from a philosophical and experiential standpoint has been carried on for many millennia. Notably, many schools of Eastern philosophical and contemplative traditions like Yoga and Buddhism have not only engaged in rigorous experiential inquiry of consciousness spanning at least a few thousand years, but also embedded elements of such inquiry into their soteriological goals. Recent work has elaborated how certain Buddhist meditation traditions may inform a study of consciousness (Lutz et al., 2007, 2008; Grabovac et al., 2011; Josipovic, 2014). However, there is currently little understanding of consciousness from the standpoint of a tradition whose initial development likely predates and influenced the historical Buddha, whose subsequent development interacted heavily with development of Buddhist contemplative practices (Bronkhorst, 1993; Kalupahana, 1994; Sarbacker, 2006; Wynne, 2007; Gray, 2016), and which continues to be alive in the present day. I refer to the Tantric tradition of Yoga^1^, which involves manipulating consciousness through meditation practice with the soteriological goal of experientially “realizing” consciousness in its “purest” or primordial essence (Gray, 2016). Here, I attempt to outline the psycho-philosophy of Tantric Yoga, and some of its associated practices used in attaining such “realization.” I use for specificity, praxis from a living Tantric Yoga tradition, namely *Ananda Marga*, whose literature also offers philosophical advances to the age-old *Samkhya* philosophy (Anandamurti, 1998) that historically formed the bases of Yoga (Bharati, 2001; Burley, 2006). Situating Tantric Yoga practices and their outcomes within both traditional philosophy and a phenomenological model of cognition, I discuss how they could inform contemporary inquiry into consciousness.

## Investigating consciousness through meditation

By most accounts, consciousness comprises the object present in consciousness (i.e., *what* one is conscious of) and the subjective aspects (the *who* that is conscious of that object) (Bliss, 1917; Bakan, 1958). As tasks for manipulating the former are relatively more straightforward, most theories of consciousness are developed to account for the object of consciousness (e.g., (Graziano and Webb, 2015; Dehaene et al., 2017; Brown et al., 2019). Recently, several authors have proposed that altered meditative states could be used to manipulate and study the subjective or subject-object interaction aspects of consciousness (Berkovich-Ohana and Glicksohn, 2014; Metzinger, 2020; Josipovic, 2021), though more clarity is needed in terms of the specific phenomenology of such states and the practices used to arrive at them.

There have been a few previous attempts at elaborating specific meditative traditions/practices with regards to how they can contribute to an empirical study of consciousness. For example, Wallace (Wallace, 1999) described the phenomenology of the Buddhist tradition of *shamatha*, or concentrated meditation practices where gradually deeper concentration is described as allowing meditators to examine consciousness—particularly, its subjective and pre-reflective attributes—in a more refined manner. Subsequently, Lutz et al. (2007) detailed Buddhist meditation practices and described three broad categories of practices: (1) those that involve developing single pointed concentration on a meditated object (or, focused attention), (2) those that involve cultivating a faculty of meta-awareness not directed toward any specific object to gain insight into a background awareness upon which mental activity transpires (open presence), and (3) those involving feeling loving-kindness toward others that is not directed toward a specific individual or a group of people (non-referential compassion). Of these, they propose the second one as particularly relevant to the study of consciousness because it involves de-emphasizing to varying degrees, first, the object of consciousness, and subsequently, a subjective feeling of possessing a sense of self (Josipovic, 2014; Dor-Ziderman et al., 2016). This kind of experiential reduction is proposed to result in advanced cases in ‘non-dual’ states, where the subject-object structure of consciousness itself be eliminated. While Wallace (1999) and Lutz et al. (2007) were influenced primarily by Tibetan Buddhism, similar descriptions of the three categories of practices have also been presented from the perspective of a contemporary Theravada Buddhist tradition (Grabovac et al., 2011). These descriptions have been accompanied by studies investigating neural correlates of self-reduced or non-dual states in long-term meditators (e.g., Josipovic, 2014; Dor-Ziderman et al., 2016) and have been followed by recent theoretical work on the possibilities and implications of the idea of a non-dual or minimal consciousness on consciousness research (Metzinger, 2020; Josipovic, 2021).

So far, the use of meditation as an empirical method to study consciousness has mainly focused on Buddhist contemplative traditions (except, see Shear and Jevning, 1999). Despite the importance of manipulating and deducing aspects of consciousness in Yogic meditation traditions, little work has been done toward understanding how these schools may contribute toward an understanding of consciousness. One reason for the lack of understanding Yogic descriptions of consciousness is their heavy reliance on epistemologies that have not been efficiently translated into contemporary frameworks of cognition.

Recently, researchers have attempted to develop an understanding of how the Yogic system of philosophy (which also forms the basis for Tantric Yoga epistemology), namely *Samkhya* philosophy, could inform contemporary research on cognition and consciousness (Sedlmeier and Srinivas, 2016; Tripathi and Bharadwaj, 2021). *Samkhya* is considered to be one of the oldest philosophical systems in the world (Raju, 1985). According to *Samkhya*, mind and matter are a composite of two primordial essences. One, a ‘‘ground’’ consciousness, known as *Purusha*, which is the witnessing counterpart of mind and matter, but is in itself devoid of any mental or physical activity. And second, an operative or energetic principle (*Prakriti*) that ‘‘activates’’ *Purusha* to transpire physical and mental activity^2^.

From the perspective of conscious experience, the intuition behind the two essences, *Purusha* and *Prakriti*, is that the former accounts for the fact that there *is* consciousness at all, while the latter accounts for the contents of experience. A metaphor sometimes used to illustrate the two *Samkhya* components is that *Purusha* is the light illuminating the room while *Prakriti* accounts for the contents of the room. *Prakriti* is proposed to further comprise three qualifying principles or *gunas*, namely, *sattva*, *rajas* and *tamas* that activate *Purusha*. *Sattva* is said to engender “desirable” mental qualities like equanimity, compassion, benevolent intellect, and spiritual curiosity. *Tamas*, at the opposite end, is said to make the mind dull, inactive, or lazy. *Rajas* is said to make the mind active, both in a positive (i.e., *sattvic*) sense of a drive to pursue one’s life purpose and a negative (*tamasic*) sense of anxious or uncontrollable mental activity. *Rajas* is thus considered as positioned between *Sattva* and *Tamas*. Each individual’s mind is theorized as a collection of the three *gunas* in varying degrees. In the historical *Samkhya* model of the mind elaborated by Sedlmeier and Srinivas (2016), the mind is functionally composed of three elements; a ‘sense mind’ that perceives sensory information, the ‘ego mind’ that attaches the perception to the individual’s sense of self, and the ‘intellectual mind’ that reacts to the information. They also mention that the purpose of a *yogi* is to make the mind gradually more *sattvic* through meditation, though it is not clear how this process may be achieved. More broadly, it is also not currently clear if and how Yogic meditation states inform the *Samkhya* framework and whether such a framework in turn informs consciousness studies? In other words, is the use of the *Samkhya* framework for Yogic meditation simply historical baggage from a time when philosophical understanding lacked present-day sophistication, or is there something about specific meditation states that phenomenologically conforms to the *Samkhya* model in a way that would make it relevant for contemporary theories of consciousness? In the following sections, I address these points in light of a living Tantric Yoga tradition, namely *Ananda Marga*. I choose this specific tradition because *Ananda Marga*’s preceptor, Shrii Prabhat Ranjan Sarkar (also known as Shrii Shrii Anandamurti)^3^, offers theoretical developments to the Yogic *Samkhya* model that map it to specific structures of consciousness, along with laying down precise meditation practices to induce states that purportedly make these structures of consciousness amenable to experiential reduction^4^ and thereby analytical deduction (Anandamurti, 1970, 1998).

## Defining Tantra and Yoga

I first clarify the terms Tantra and Yoga in the context of this paper as they are often used in a variety of different ways (Gray, 2016; Mallinson and Singleton, 2017), most of which are not relevant to this paper. The terms *Tantra* and *Yoga* can be traced back to written texts from at least fifth century CE (Gray, 2016) and fifth to third century BCE (Flood, 1996), respectively; though oral traditions refer back to much further back in time (Mallinson and Singleton, 2017; Bjonnes, 2018).

Vastly different traditions and systems of practice come under the broad category of Tantra (White, 2000; Gray, 2016). However, from the perspective of meditation practices (especially in relation to the exploration of consciousness), the common feature of these practices is contemplating something subtle (like the idea of a transcendental or a non-local consciousness) through a relatively gross or embodied form (e.g., a deity/Guru, a geometric pattern or *yantra*, a sound or *mantra*). In expounding *Ananda Marga* tantric practices, Sarkar offers a definition of Tantra based on its Sanskrit etymology (Anandamurti, 1994b). Sanskrit is an ancient language whose word constructions are an amalgamation of root sounds or words. Thus, by deriving the definition of *Tantra* from its linguistic roots, Sarkar intends to imbue *Tantra* with a primordial semantic essence that may have been lost in its transformation through the ages. The word *Tantra* is etymologically derived in two ways. One, where the word root *ta*(*m*) means mental staticity (much like its use in the word *tamas*, above), and second from the word root *tan*, which means expansion. In both cases, the word root *tra* means emancipation, or release from. According to Sarkar, *Tantra* is thus a system of practices that liberate the mind from mental staticity or that help expand the mind to the point that it is liberated from “mundane bondages” (Anandamurti, 1994b).

The word Yoga also appears in traditional texts with multiple definitions (Mallinson and Singleton, 2017; Bjonnes, 2018). Probably the most popular definition is the one appearing in the classical Yoga Sutras of Patanjali, dated between 400 BCE and 300 CE. This definition states, ‘*Yogas chitta vrtti nirodhah’* (Bharati, 2001; Desmarais, 2008; Bærentsen, 2015), roughly translating to, Yoga is the ability to control mental fluctuations. Another definition that appears in a later text called the *Jnana Samkalini Tantra*, states ‘*Sarvachintaparityago nishchinto yoga uchyate*’ (Prajnanananda, 2010), or Yoga is a state where the mind is free from all thoughts. A third definition of Yoga is *‘Samyoga yoga ityukto jiivatmaa Paramatmanah*’ or Yoga is the unification of individual consciousness with Cosmic Consciousness (i.e., individual Purusa or *jiiva* with the Cosmic Purusa or *Parama Purusha*)^5^. This definition is thought to have first appeared in the text *Yoga Yajnavalkya*, estimated between 2nd century BCE and 4th century CE (Divanji, 1953; Mohan, 2000), though Sarkar attributes its existence to oral traditions much longer (Anandamurti, 1994a).

In his exposition of Yoga praxis, Sarkar, utilizes this third definition of Yoga, which he suggests is the one that most conforms with the etymological essence of the word Yoga (Anandamurti, 1994b). According to him, the word Yoga is derived from the Sanskrit root *yunj*, literally meaning union or unification (which are also derived from a similar Indo-Latin root). Moreover, this third definition corresponds to a non-dualistic interpretation of *Samkhya* in contrast from Patanjali’s dualistic view (Schweizer, 1993; Tripathi and Bharadwaj, 2021), which conforms with Tantric styles of Yoga (Anandamurti, 1970, 1998)^6^.

## An updated *Samkhya* model of cognition

As mentioned above, in traditional *Samkhya* philosophy, the action of *Prakriti* on *Purusha* leads to the intellectual, ego, and sense mind (Sedlmeier and Srinivas, 2016; Tripathi and Bharadwaj, 2021). Here, *Purusha* is the underlying “ground” consciousness upon which all mental activity transpires. In this sense within conscious experience, the mind is defined as any kind of expression, movement or activity upon *Purusha* (this is a critical point of difference between the *Samkhya* model of cognition and contemporary cognitive psychology, where in the latter everything within conscious experience would be considered mind).

**FIGURE 1:**
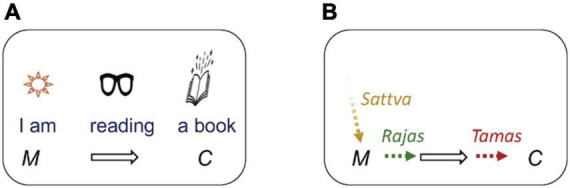
**(A)** The experience of “I am reading a book” is subdivided into the mental object (“book”) or *citta* (depicted by C), the mental action performed on that object (“reading”) or *ahamtattva* (depicted by the black arrow), an existential component of consciousness (“I am”) or *mahattattva* (depicted by by M). Finally, the component “I am” is proposed to further comprise an experiencer (“I”) and a bare experience (“am”) devoid of the mental act and mental object. “I” is proposed as the unqualified background field or essence consciousness upon which all experience takes place depicted by the background encompassed by the rounded rectangle. **(B)** The three *gunas, prakriti, purusha, samkhya* gunas of Prakriti according to Samkhya philosophy qualify the background essence consciousness (equated to Purusha or witnessing consciousness in Samkhya). *Purusha* (background) qualified by *sattva*(*guna*) begets *mahattattva* (M). *Mahattattva* qualified by *rajas* begets *ahamtattva* (black arrow). *Ahamtattva* qualified by *tamas* begets *citta* (C).

In his philosophical works, Sarkar further builds upon the *Samkhya* model by proposing that the three *gunas* influence *Purusha* to engender a hierarchy of structures of consciousness from subjectivity to objectivity. To illustrate this, he considers how an awake conscious experience presents itself to the first-person as expressed in their verbal description of it, and divides it into different experiential subcomponents (Anandamurti, 1998). He considers as an example, the statement “I am reading a book” (Figure 1A). Here, ‘‘book’’ is the object of consciousness. Sarkar describes the mental object as the ‘‘shape’’ taken by the ‘‘object portion’’ of the mind, which he labels *citta^7^* . The mental act of ‘‘I am reading,’’ and in general any mental action, he attributes to *ahamtattva* or ego portion of the mind, what he sometimes calls the ‘‘doer I’’ (*aham* literally means ego, and *tattva* means essence). Next, he proposes that implicit within the mental action is the subjective quality of being someone who in turn performs the action. This existential feeling is immanent in the phrase ‘‘I am,’’ where the subject of experience possesses a feeling that ‘‘I exist irrespective of what I do’’ (i.e., irrespective of the action performed by *ahamtattva*). Sarkar calls this aspect of the subject, *mahattattva* or the portion of the mind that gives an individual an ‘‘I’’ feeling or a feeling that ‘‘I exist’’ (*mahat* means great). Finally, he proposes that there is a further sense of phenomenological duality inherent in the conscious feeling of ‘‘I exist,’’ where ‘‘I exist’’ or ‘‘I am’’ comprise two separate structures of consciousness; one an unqualified ‘‘I’’ and second the property that qualifies the ‘‘I’’ into experiencing itself. In other words, implicit within *mahattattva* (or ‘‘I am’’) is a pure essence of ‘‘I,’’ which he also calls the witnessing consciousness or the ‘‘knower I’’ (i.e., the ‘‘I’’ that knows that ‘‘I exist’’) that is transcendental to mental activity. The reason he considers this ‘‘I’’ as transcendental is that it is devoid of even basic self-reflectivity and is thus not accessible as a conscious experience (and in that sense could be considered metaphysical or metempirical). It is upon this non-reflectable ‘‘I’’ that all mental experience (including the reflected sense of ‘‘I am’’) is said to transpire. To summarize, according to this framework, a conscious experience comprises three potentially dissociable aspects of the subjectivity -- a pure witnessing consciousness, a sense of existential feeling to the individual, the doing portion of the mind - in addition to the mental object^8^.

Sarkar then connects the above dissociated structures of consciousness to the *Samkhya* framework (Figure 1B). He equates the witnessing “I” to *purusha* (also commonly known as *atman*). He proposes that *mahattattva* or the “I exist” aspect of the mind emerges by the activity of *sattvaguna* upon *Purusha* (specifically, by the metamorphosis of *purusha* by *sattvaguna* into *mahattattva*). Next, the activity of action-oriented *rajoguna* (the conjuncted word for *rajas* + *guna*) upon *mahattattva* results in the *ahamtattva*, the doing aspect of the mind. Finally, the activity of the static *tamoguna* (*tamas* + *guna*) upon *ahamtattva* results in *citta* or the mental object, which by nature is static. Through this formulation, he provides a systematic cognitive extension to the *Samkhya* framework.

## Meditation as means to eliminate different levels of experiential dualities

Sarkar’s philosophy outlined above implies a systematic dissociation of the components of consciousness culminating in what is considered the transcendent. The first-person experience of dissociating these components in consciousness is said to have transformative existential implications to the experiencer and in their relationship to others, central to the Tantric Yoga goal of self-realization^9^. Specifically, the three mental cognitive structures--namely the mental object (*citta*), mental doership (*ahamtattva*), and the existential aspect of mind (*mahattattva*)--induce different types of differentiations and dualities in consciousness. These differentiations within consciousness prevent the realization of the ‘‘true essence’’ of consciousness, which is free from all ‘‘bondages’’ and suffering, and is by nature transcendental and ‘‘infinitely blissful’’ (*ananda*)^10^. The differentiations and dualities are said to be reduced and gradually eliminated through a systematized set of meditation practices (known as *sadhana*) leading the practitioner to the undifferentiated or non-dual stance of consciousness (*purusha*). In his seminal 1955 work, Ananda Marga: Elementary Philosophy, Sarkar describes how the three stages of differentiation are eliminated stepwise (Anandamurti, 1998):

“… *the preliminary saìdhanaì (intuitional practice) has to be carried out by the consciousness metamorphosed as citta, by which this projection of consciousness retracts into ahamìtattva. This leaves only ahamìtattva and mahattattva. So, the next entity to carry out saìdhanaì is the consciousness metamorphosed as ahamìtattva. It is to free itself from the qualifying influence of the principle of Prakrti creating it, by its dissolution into mahattattva. Thus, only mahattattva or pure feeling of “I” remains. This is the stage of savikalpa samaìdhi where only mahattattva or pure “I” feeling indistinguishable from the Cosmic “I” remains. After this, mahattattva carries out saìdhanaì and dissolves itself in the unit consciousness completely, freeing consciousness of the qualities imposed by the influence of Prakrti. It achieves emancipation from the bondage of Prakrti, and that is called nirvikalpa samaìdhi. Thus, the saìdhanaì or intuitional practice that human beings have to carry out begins with citta, to be followed by ahamìtattva and finally by mahattattva, which emancipates consciousness completely from the qualifying influence of Prakrti.*”

In other words, according to Sarkar, the first stage of *sadhana* involves suspending or “converting” the *citta* into *ahamtattva*. The second stage involves converting the *ahamtattva* into *mahattattva*. And finally, *mahattattva* into *purusha*. Additionally, in this passage, he also offers a cognitive definition for what are historically considered the two most advanced states of absorption in Yoga, namely *savikalpa* and *nirvikalpa samadhi* (Bharati, 2001). I elaborate on this in the next section about praxis.

## The praxis of Tantric Yoga

### The eight “limbs” of Yoga

Despite the large number of historical and extant schools of traditional Yogic practice, most (including Tantric Yoga) share a common underlying framework of practice known as *Ashtanga Yoga* (or the eight limbs/organs of Yoga), first believed to have appeared in the Yoga Sutras of Patanjali (Bharati, 2001).

The eight limbs of Yoga in order are, *yama*, *niyama*, *asana*, *pranayama*, *pratyahara*, *dharana*, *dhyana*, and *samadhi*. *Yama* and *niyama* consist of five ethical principles each to guide a yoga practitioner’s conduct in relation to others and themselves, respectively. In most traditions of Yoga, the teacher (or *guru*) maintained a close watch on the conduct of the disciple before initiating them into the subsequent limbs. In the *Ananda Marga* practices as well, progress in meditation is considered inextricably linked to the extent one is able to follow *yama* and *niyama*.

*Asana* refers to the practice of yogic postures. This is arguably the most well-known practice in Western adaptation of Yoga (specifically Hatha Yoga) to improve physical health. However, in traditional accounts, *asanas* have two purposes: (1) preparing the body for meditation (*dhyanasana*), and (2) improving physical/mental health (*svasthyasana*) (Anandamurti, 1991). *Pranayama* is the practice of controlling respiration. By sufficiently controlling the breathing rate, a meditator is said to be able to concentrate better (Anandamurti, 1991).

The word *ahara* literally means food in Sanskrit but in the practice of Yoga it implies mental fodder or mental content. *Pratyahara* is the practice of withdrawing the mind, as much as possible, from mental content that distracts one’s meditative concentration. This involves procedures for gating out both sensory content from the external world as well as distractive thoughts. The mind withdrawn from distractions is then directed to concentrate on the chosen object of meditation (hereon, the meditand). The practice of concentrating on the meditand is known as *dharana*.

While *dharana* is concentration on a mental object, the word that translates to meditation is the next limb, *dhyana*. The difference between *dharana* (concentration) and *dhyana* (meditation) is an important distinction that many Yogic masters have tried to expound upon. The most commonly described difference is that *dhyana* involves an unbroken flow of consciousness upon the meditand (Vivekananda, 1915; Bharati, 2001; Sarbacker, 2006). On the other hand, *dharana* would be more of a sustained effort to focus on the meditand with occasional distractions breaking concentration. An alternative way to conceive this difference could be an effortful versus effortless state of attention (Bruya, 2010).

Sarkar expounds on the distinction between *dharana* and *dhyana* in two discourses from 1957 (Anandamurti, 1988c) and 1964 (Anandamurti, 1988d), as follows:

“*the citta takes the forms of the image or sensation which is carried to it with the help of the sensory nerves*… *There is always a gap between two successive images, but due to the rapid succession of the images, the gap is not perceived*… *Since, the sensations of the external object are not continuous, the image in the citta is also not continuous. Thus, Dhaìranìa is not dynamic, for individual images which are formed on the citta, are all static and will not remain unless immediately followed by another image*…

*The object in dhyaìna is always internal and so citta can take its form without the help of any external sensations. When there is no necessity of external sensations, there is also no gap between one sensation and another; and the form which the citta adopts in dhyaìna is continuous*…”

“… *when you try to hold something external within your mental world it is called Dhaìranìaì. So in Dhaìranìaì, there is a static force. But when something is moving and that movement has been accepted by you as it is, it is called Dhyaìna. So in Dhyaìna there is a dynamic force. Dhyaìnakriyaì is just like a thread of molasses. When poured a thread is created; there is force, there is movement in that thread but it appears to be something static.*”

According to the first quote, because *dharana* involves holding an external sensation that is non-continuous—consistent with recent cognitive science literature on discrete perception (VanRullen, 2016)—consciousness during *dharana* consists of discrete “static” mental objects. On the other hand, *dhyana* involves holding an internal object, which when done skillfully allows consciousness to take upon a “dynamic” or unbroken or continuous character. Moreover, the second quote appears to imply that unlike *dharana*, *dhyana* may not just involve stilling the mental object but the very process of meditating on the object (analogous to the apparent staticity of the flow of molasses beyond just the stationary molasses already poured in a container). This may be understood through the phenomenological concept of intentionality, according to which a conscious experience is said to involve a mental act originating from the subjective “pole” of experience and is directed toward the mental object (Husserl, 1998). While *dharana* involves stabilizing the mental object, according to the above description, *dhyana* may additionally involve a sort of “objectification” and stabilizing the mental act.

The word *samadhi* comprises *sama* + *dhi*, where *sama* is etymologically a cognate of the word “same” and *dhi* means flow of mind or flow of consciousness. *Samadhi* can thus be roughly translated as the sameness or unchangingness of experience. *Samadhi* is considered the culmination of Yoga practice and is attained by the “perfection” of *dhyana* (e.g., Sarbacker, 2006). *Samadhi* can be considered as a sort of trance state where meditation is perfected to the degree that it no longer remains a mental activity within consciousness (i.e., in the sense of the verb ‘to meditate’) but becomes a state where consciousness is said to be fully encompassed by the meditand. In other words, s*amadhi* would involve complete suspension of the separation of experience into mental act and its object. One way this suspension could be achieved is by eliminating the mental act, leaving in consciousness only the mental object. However, a distinct feature of Tantric Yoga is that the object of meditation is often not just purely sensory, but one that supplements sensory qualities (such as, internal sound of a silently repeated mantra, an imagined visualization, etc.) with an idea embodied by those qualities. This is in contrast to non-Tantric styles of meditation like TM where the mantra, as the meditand, is taught to lack meaning (Shear and Jevning, 1999). The idea associated with the object is said to determine the mental qualities ultimately engaged and suspended in a particular type of *dhyana* and its ensuing *samadhi*. Accordingly, Yogic (and Buddhist) literature mentions many different types of *samadhis* (Anandamurti, 1988b,^1993^; Sarbacker, 2006; Tripathi and Bharadwaj, 2021). I will return to these points on *dhyana* and *samadhi* later.

### Stepwise elimination of experiential differentiation

As mentioned earlier, *Ananda Marga* meditation practices are formulated within a framework where consciousness is composed of three reducible components that constitute the mind (*citta*, *ahamkara*, *mahattattva*), which when experientially reduced are believed to lead to the irreducible (and thus transcendental) consciousness (*purusha*). The meditation practices are thus designed to reduce mental constituents of consciousness leading to the irreducible stage.

**FIGURE 2:**
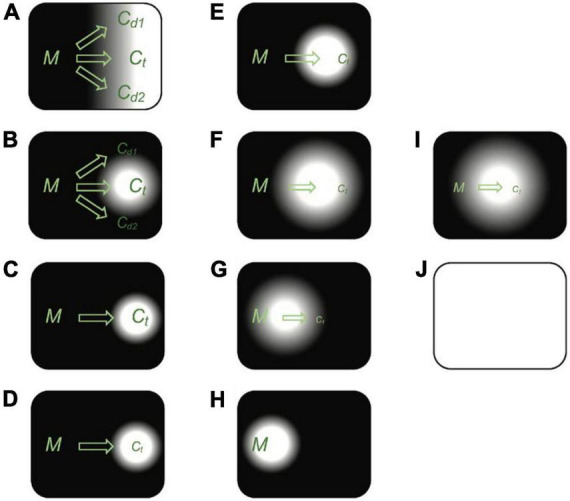
A depiction of the variety of Tantric Yoga meditative states of consciousness. The shades of white and black depict the components of consciousness that are salient and pre-reflective, respectively, to the first-person. The different sets of panels in the three columns are meditation states arrived through reduction of *citta*, *ahamtattva*, and *mahattattva*, respectively. **(A)** At the beginning stage the mental object includes several distractions (Cd1, Cd2, …) along with the meditative target (Ct). The target along with distractions are thus salient along with a feeling of doership, agency, or ownership of one’s mental actions on the mental object. This is depicted by white Cs and partly white arrows. **(B)** Concentrative practices (*dharana*) enable selective enhancement of Ct’s saliency. **(C)** Mental withdrawal (*pratyahara*) practices diminish distractions, making Ct even more salient. **(D)** Continuous sustained attention on Ct makes it qualitatively more subtle, almost as if receding into a blank background. **(E)** With citta reduced, the feeling of the act of meditating becomes salient – illustrated by the leftward movement of the white spot encompassing more of the arrow. **(F)** The mental act and object start to diminish within consciousness – illustrated by the smaller size of the arrow and Ct. **(G)** Almost at the same time as **(F)**, there is a conscious experience with a diminished sense of the act of meditation upon the mental object – illustrated by the white spot moving leftwards to cover M. **(H)** Mental act and object are eliminated from consciousness with only a bare existential consciousness remaining – *Savikalpa Samadhi*. **(I)** Phenomenological attempts to suspend all aspects of conscious experience including the mental act, mental object, and a sense of existentiality, into an idea of a witnessing consciousness transcending experience. **(J)** All experience suspended from consciousness – *Nirvikalpa Samadhi*.

Each *Ananda Marga* meditator can learn up to six “lessons” of Tantric Yoga meditation (also known as *Sahaja Yoga* or *Rajadhiraja Yoga*) from an ordained instructor, known as an *acharya* (Anandamurti, 1987; Taraka and Acyutananda Avadhuta, 2014)^11^. The lessons are taught gradually as the instructor ascertains that the meditator has made progress with the previous lessons. In the following section, I describe the phenomenological approach taken within some of these lessons for reducing the different structures of consciousness. My objective here is not to go into the specifics of each lesson^12^, but instead illustrate the kind of conscious states the meditation practices purportedly enable. The different states are illustrated in Figure 2 where *mahattattva* is depicted by the *M*, *citta* by C, and *ahamtattva* by the arrow connecting the two. *Purusha* is not represented by a letter but as the background upon which the three mental structures are expressed. The whitened portion within each panel reflects the component that is most salient in consciousness during a state while the dark portions are pre-reflective. Note that unlike models of cognition where arrows between hierarchies are bidirectional, the arrow here reflects the phenomenological flow of conscious experience like in intentionality (Husserl, 1998).

#### Step 1 – Reducing citta

The scope and variety of possible mental objects is vast, including all sensations, perceptions, concepts, and thoughts. This large variety of what an individual experiences in their consciousness is efficiently summarized by enactive (or “4-e”) accounts of the mind (Newen et al., 2018). According to enactive accounts, an individual’s experience in the world is intrinsically related to their needs and actions in the world, their bodily constitution, and their embeddedness and extension into their social and physical environment (such an account elegantly encompasses both the biological evolution of an organism as well as the developmental trajectory of a specific individual). Due to the ever-changing nature of an individual’s actions and needs, the object of consciousness is constantly busy and ever-changing over time. I illustrate this in Figure 2A where *C* is divided into *C*_*t*_—the target object of meditation—and *C_*d*1_*, *C_*d*2_*—for distractions. At the beginning of meditation, the target and distractive components of *citta* have relatively similar saliency within the meditator’s consciousness. There may also be a faint awareness of the mental act in the form of a sense of agency or ownership of one’s mental actions (Reddy et al., 2019, 2020) (similar to the idea of a “minimal self” in cognitive science, Gallagher, 2000). In Figure 2A, this is depicted as a gradient from black to white starting from the arrow (representing agency) to multiple different *C*s (representing the high saliency of distractors).

One set of methods for silencing mental fluctuations in *Ananda Marga* meditation practices involves cultivation of concentration (*dharana*). This includes lessons for practicing breath control (*pranayama*) and training single-pointed concentration. These methods enable better selective application of attention on the target of meditation (Figure 2B).

Another set of methods for reducing the *citta* involve practices for eliminating distractions by restricting the enactive range within which the *citta* can vary. These methods constitute mental withdrawal (*pratyahara*) and are operationalized in two steps (Hewitson, 2014). The first step takes into account that the human mind has evolved and developed on this planet and is embedded in an individual’s physical and social environment. It thus involves internal visualization for helping the meditator dissociate themselves for the meditation period from their physical and social environment, allowing them to face their own consciousness in isolation^13^. The second step takes into account that the experience of having a body (1) contributes to distractions from bodily sensations (e.g., aches, itches, etc.), and (2) plays a major role in engendering a sense of self within the bounds of one’s body (Seth, 2013; Allen and Friston, 2018). This step involves internal visualizations to help the meditator withdraw their mind from the physical body. For most practitioners, these two practices may take years or decades to perfect. However, the emphasis at this stage is to be able to reduce the influence of embedded and embodied factors rather than eliminate them completely. The ensuing mental experience, less influenced by worldly and bodily factors (Figure 2C), is then ready to be focused on a meditand that is neither in the world nor in the body (at least theoretically for a beginner). Here, the object of meditation is typically a *mantra*, an associated visualization, and a meaning that connects the two (I discuss more about the meaning in the next subsection).

The final step in the reduction of *citta* occurs through the maintenance of concentration on the meditated object. As concentration is maintained, there is a sense of “fading away” of the experiential solidity of the sensory components of the meditated object (Figure 2D). As a result, the same sensory object appears in a more subtle or refined form with lesser effort. A similar process of “letting the mantra fade away” is also described in other practices (e.g., Shear and Jevning, 1999). Such a process may be analogous to sensory adaptation, which can lead to near elimination of unchanging stimuli from conscious awareness (Martinez-Conde et al., 2004).

#### Step 2 – Reducing ahamtattva

As the object of consciousness (*citta*) gets somewhat reduced within experience, what tends to become prominent in conscious experience is a feeling of ‘being someone’ (regardless of the sensory/cognitive content which that ‘someone’ experiences). This (initial) subjective feeling of ‘being someone’ typically involves a sense of ego or agency in the sense that “I am meditating (on the meditand)” (Figure 2E). This sense of ego is posited to carve an individuality within consciousness that enables one to think that they are separate from others and the world in the sense that the individual is the one performing the actions (and not that the actions are being performed within the context of the existence of the world as a whole). According to Sarkar it is this ego aspect of the mind that is engaged in a process of receiving enjoyment and suffering from the world (Anandamurti, 1998). This “self-other duality”—i.e., separation between one’s feeling of an individual selfhood that acts in relation to the outside world—has parallels in contemporary cognitive science where the self in its affective and individual form is posited as a mental construct that is in a closed loop enactive/predictive relationship with the world (Seth et al., 2012; Hohwy, 2015; Allen and Friston, 2018); similar predictive models of action are also used to explain a sense of agency. As in many other meditative traditions, this self-other duality is considered an “imperfect” stance of consciousness within Tantra Yoga. The imperfection is multiple senses: (1) in that the individual does not perform actions isolated from the world but in interaction with it (and by virtue of its very existence), (2) in that the sense of ego entails affective states that are temporary in character and having the potential to lead to suffering, and (3) in that they “hide” a “purer” non-dual/transcendental consciousness beyond such temporary states where the world is not experienced as different from oneself that can serve as a self-actualizing or spiritual ideal.

As self-other duality is linked to performing mental actions upon the world, the objective of meditation at this stage is theoretically framed as suspending or converting *ahamtattva* (doer ‘‘I’’) to *mahattattva* (existential ‘‘I’’). The same objective is also expressed by the meaning of the *mantra* during meditation^14^. To achieve this objective practically, the meditator is taught to initiate a *dhyana* in the form of a phenomenological shift with regards to the meditand. The shift involves conceiving of the meditand not just as the part of the mind that is the object of meditation (i.e., the *citta*), but *citta* combined with the doership (*ahamtattva*). In other words, the meditator now tries meditating upon a combination of the following as the new meditand, (1) whatever is left of the reduced object of meditation (i.e., *citta*), and (2) oneself as an individual performing meditation (i.e., *ahamtattva*). The objective of the meditation here is to reduce *ahamtattva* to *mahattattva*. Another way this process is instructed to the meditator is that they take the (combined) meditand as an “object” to an existential consciousness *(mahattattva)* as its subject while “allowing” such an object to merge into such a subject. Here, existential consciousness is explained phenomenologically as the residual part of their conscious experience from which an individual sense of ego or doership has been fully eliminated. When such a *dhyana* is initiated, it is not expected that the meditator would immediately arrive at a state where the actual sense of ego can be fully objectified or amenable to suspension (unless perhaps in a highly advanced meditator). Rather, at the beginning, the idea that one’s *ahamtattva* is the object of *mahattattva* could itself be considered a conceptual form of *citta* (i.e., as a mental object, and not actually *ahamtattva*). In other words, the *dhyana* starts off as a schema or an idea about the state one is expected to attain. As the meditator proceeds through practice, they are supposed to use this schema of merging the individual ego/selfhood to a non-individualized existential aspect of consciousness (*mahattattva*) recursively with the skill of unbroken flow of consciousness (*dhyana*) to enter gradually deeper states where the sense of doership (*ahamtattva*) is diminished thus making meditation gradually more effortless (Figure 2E). Recall that according to Sarkar’s definition of *dhyana*, a meditator ought to stabilize the mental act of meditation upon the mental object in a way that it appears static or still within consciousness. Such a phenomenological approach might make it amenable to a process similar to sensory adaptation but at a higher level of the cognitive hierarchy (i.e., involving the combined *citta* and *ahamtattva* as meditand). A momentary entry into such an experience of diminished *ahamtattva* may be reflected in reports by meditators of the kind “the meditation was done through me” (i.e., in my *mahattattva*) rather than “I was meditating” (as an agent and owner of the act of meditation). At the same time, a feeling of being conscious devoid of doership/ego (*mahattattva*) appears to become salient in consciousness (Figure 2H). During this process, meditators may start reporting experiences like emptiness of (contents of) consciousness, an expanded sense of space and time, limitlessness of consciousness, everything as consciousness, or a feeling blissfulness beyond mundane joys and pleasures (Piron, 2001; Gamma and Metzinger, 2021; Katyal and Goldin, 2021a; Maxwell and Katyal, 2022). With yet more skill, a meditator may also be able to momentarily attain a state of mental stillness where all mental action is entirely suspended (Figure 2G). Finally, in rare, advanced cases, a meditator is said to be able to achieve the state where neither *citta* nor *ahamtattva* remain active and all that remains is bare *mahattattva*, a non-reflexive feeling of being conscious, or the experiential state of “pure” consciousness (Figure 2H; Anandamurti, 1998). This state is known as *Savikalpa Samadhi* (*Sa* = with, *vikalpa* = concept or thought or feeling) and is anecdotally reported as involving the bare feeling of being a “blissful infinite consciousness” (Yogananda, 2005, Chapter 26; Pranavatmakananda, 2017, Chapter 18).

As *ahamtattva* is framed as an enactive “product,” and is inextricably tied to an individual’s otherwise strong beliefs about agency and ownership of mental and physical activity, its release of consciousness is not framed as an easy task (unlike self-transcending states from other traditions like TM, Shear and Jevning, 1999). Moreover, stronger or “thicker” such beliefs in an individual (Shoemaker calls properties that make an individual *that particular individual* “thick”; Shoemaker, 2011), the more meditation practice it would take to suspend *ahamtattva*. Thus, the state attained during meditation is also considered closely linked to one’s everyday behavior and how ‘‘strongly’’ a sense of agency and individuality is experienced during such behavior^15^.

#### Step 3 – Reducing mahattattva

The above-described meditation practices for reducing *citta* and *ahamtattva* are part of the ‘‘first lesson’’ of *Ananda Marga* Tantra Yoga (also known as *pranidhana* or surrender of ego). Reduction of *mahattattva* occurs through the sixth (and final) lesson^16^ (also known as *anudhyana* or approaching transcendental consciousness in meditation). During a sitting session of meditation, a meditator typically performs the sixth lesson at the end. This is because for the sixth lesson, it is assumed that the meditator has already been able to clear the mind off most distractions and has entered a sufficiently deep state of meditation to start off.

As mentioned earlier, in Tantric Yoga philosophy, even the experience of pure consciousness (or minimal phenomenal experience; Metzinger, 2020) is considered to entail a duality between bare experience and its experiencer. In other words, the ‘‘purest’’ form of consciousness is not pure experience but (a postulated) transcendental source of such experience. The sixth lesson can thus be considered to begin with a postulation. The postulation is that in the absence of bare experience, consciousness assumes an unqualified stance of a proto, primordial, or essence consciousness (*purusha* or *atman*)^17^. If such a postulation is not agreeable to the meditator, they will not be initiated into the sixth lesson (and can continue with previous lessons). However, often the previous five lessons tend to induce gradually subtler blissful experiences in the meditator (Maxwell and Katyal, 2022) building a curiosity about the possibility of a “transcendental blissful consciousness” by the time they arrive at the sixth lesson of meditation.

Derived from the postulation of a protoconsciousness, the phenomenological approach of the sixth lesson is as follows. The meditator again starts by either reducing the *citta* and *ahamtattva* to the extent they can using above-described approaches or continuing from such a state after having done earlier lessons. Then through the aid of certain visualizations, the meditator induces a *dhyana* that their entire conscious existence is witnessed by a transcendental consciousness. According to Sarkar, even though transcendental consciousness is the “supreme subjectivity” and cannot be an object of the mind (Anandamurti, 1978), this kind of a phenomenological attitude of “objectivizing subjectivity” (through a mental idea of transcendental consciousness) is assumed at the start of meditation to subsequently allow oneself to “subjectivize this objectivized subjectivity” (i.e., “becoming” transcendental consciousness). Once this attitude is assumed, it is followed by repeated phenomenological “attempts” to let one’s entire conscious experience be submerged or “surrendered” into an idea of a non-qualified transcendental consciousness completely beyond or devoid of conscious experience (meditators are provided more specific instructions on how to achieve this). Importantly, according to Tantric Yoga, such complete submergence is not possible unless the motivation of the meditator (Reddy and Roy, 2019) to go into the non-qualified (or *nirguna*) state is extremely strong (Anandamurti, 1994b). The motivation of the meditator to go from the qualified to the non-qualified stance of consciousness is termed within different schools of Yoga as *nirguna bhakti* (love of the non-qualified), *parabhakti* (love of transcendental subjectivity) or *kevala bhakti* (love of the state of “only-ness”) (Anandamurti, 1988a,1994b). The phenomenological approach of trying to enter the non-qualified state is sometimes likened to jumping into an infinite chasm where one will lose all identity and all experience in favor of the source of consciousness. Making that jump would thus entail a strong motivation by the meditator to do so, in the absence of which, the non-qualified state would remain out of reach even to an advanced meditator. This final non-qualified stance of consciousness is known as *Nirvikalpa Samadhi* (*nirvikalpa* = without *vikalpa* or without experience) or the state of *kaivalya* (only-ness). According *to Sarkar* (Anandamurti, 1970, *Ananda Sutram* verse 1-24), because *nirvikalpa samadhi* is absent of experience, it is an inferred state, and the proof of such a state (i.e., its distinction from all other states) is the lingering bliss unparalleled by any other experiential state (or non-experiential state like dreamless sleep) after one returns from it. In other words, this state—despite lacking experiential awareness—is still considered a unique state of meditation where consciousness is considered in its inactivated form based on the argument of continuity of the primordial substance whose property is consciousness in experience [see Thompson (2014) for a similar continuity argument in Indian philosophy applied to inferring consciousness during sleep].

#### Co-reduction of different structures

Note that while the above stepwise manner of describing experiential reduction of structures of consciousness aid elucidation, the manipulation of the different structures through meditation is neither strictly sequential nor independent. This is in agreement with Husserl’s phenomenological principle of ‘correlational *a priori*,’ according to which the mental object and subjective feelings directed toward it are interdependent (Husserl, 1970; Gutland, 2018). For example, the reduction of the object simultaneously would “expose” the ego and existential feelings to different degrees depending on an individual’s psychosocial history. The reduction of the ego would simultaneously expose the existential feeling. At the same time, long-term meditation is associated with a general decrease in ego aspect of consciousness where a long-term meditator is said to be able to experience waking consciousness with a reduced trait sense of individualized agency or self-other duality (Josipovic, 2021).

### From state to trait

While *samadhis* are considered the objective of Tantric Yoga from a state standpoint, their attainment (including *nirvikalpa samadhi*) once or a few times is not a sufficient objective of meditation practice. In the long run, the objective of meditation is to be able to at will experience—even during a regular waking state (not formal sitting meditation)—the world not as something external to oneself but as constituted within (*ahamtattva*-reduced) consciousness and existentiality as constituted within a (*mahattattva*-reduced) transcendental realm that one can gain “access” to. This type of trait alteration through perfecting meditation states is sometimes known as *sahaja samadhi* (or easy samadhi; easy in the sense that one easily or rapidly accesses the experiential attributes of samadhi). While, a thorough development of sahaja states is beyond the scope of this article, as mentioned above, such an idea could be developed as trait reduction of ego aspect of consciousness in a manner similar to what has been described as non-dual awareness in a recent work (Josipovic, 2021).

## Discussion

I offer a joint traditional, cognitive, and a praxis-based account of Tantric Yoga meditation states. I do so by first using traditional *Samkhya* philosophy and its recent developments by Sarkar to arrive at what may be considered a theoretical phenomenological dissociation of structures of consciousness into the mental object (*citta*), the sense of performing a mental act upon the object (*ahamtattva*), an existential feeling invariant of the mental act and object (*matattattva*), and a proposed meta-empirical transcendental stance (*atman*) (Anandamurti, 1998). I then offer an account of the *Ananda Marga* Tantric Yoga practices that are used to translate this theoretical dissociation into a practical dissociation to validate the theory in the first-person. I briefly discuss the implications of the present work on consciousness research.

*Citta* and *ahamttatva* have clear parallels in contemporary consciousness studies. *Citta* can be considered synonymous with the contents of consciousness, which include both sensory (exteroceptive, interoceptive, or proprioceptive) and conceptual (thoughts, memories, emotions) objects. *Ahamtattva* includes among other features a sense of agency and ownership in relation to one’s actions, body and mental contents in general. Both topics are extensively studied in cognitive science. Recent work moreover relates the two by positing the sense of agency or ownership as a higher-order cognitive process making (Bayesian) probabilistic predictions about (lower-order) sensorimotor contingencies (Hohwy, 2015). When it comes to the contents of consciousness, current research paradigms typically operate by manipulating the availability of sensory stimuli to perceptual awareness — i.e., when stimuli that become perceptually aware versus when they do not (e.g., backward masking, threshold detection, or visual illusions such as binocular rivalry). However, in such paradigms even when a subject lacks awareness of a sensory stimulus, it is not as if they are not aware of anything at all; they would, for example, still be aware of the background upon which the stimulus is presented. In other words, there is still an object present in consciousness, like the stimulus background or mask. Such paradigms thus tacitly assume that consciousness is equivalent to the object in consciousness and do not address the possibility of reducing (or removing) objectivity itself from consciousness. In recent years, several researchers have proposed the use of meditation-induced states of consciousness to investigate an “objectless” awareness (Lutz et al., 2007; Josipovic, 2014; Vago and Zeidan, 2016; Srinivasan, 2020), “awareness of awareness” (Gamma and Metzinger, 2021; Sparby and Sacchet, 2022), “pure consciousness” (Metzinger, 2020) or “consciousness as such” (Josipovic, 2021). The present work contributes to this literature by outlining a traditionally inspired cognitive framework for such states, which in turn enables an understanding of the heterogeneity of them. For example, the present work suggests that it may be appropriate to label such states in a graded manner as “object-reduced states” (e.g., Figures 2D–H) with very specific potential states being truly objectless [see Josipovic (2021) for how different objectless states may be phenomenologically approached by different meditation techniques]. Such an approach could potentially help achieve common ground and allow us to make sense of states from different traditions in relation to how they inform consciousness studies. A more practical consequence of this idea of object-reduced consciousness is that studies of such states would be better of using continuous qualitative scales (Dor-Ziderman et al., 2016; Katyal and Goldin, 2021a) rather than treating them as categorical (i.e., with object vs. objectless) as is done in neuroimaging designs using block-based contrasts (e.g., Josipovic, 2014; Winter et al., 2020). As with previous work on other meditation techniques (Lutz et al., 2019; Pagnoni, 2019), practices involving reduction of *citta* and *ahamtattva* could be understood mechanistically in terms of active-inference-based accounts of predictive processing. According to such accounts, higher-order cognitive processes are proposed to generate predictions about lower-order sensorimotor contingencies and the mismatch in such predictions act as signals that impel an agent to act upon the world to improve the accuracy of future predictions (Friston, 2010). Experiential co-reduction of *citta* and *ahamtattva* may work by withholding top-down propagation of predictions through so-called “meditative non-action” (Pagnoni, 2019). Our recent study offers a promising step in investigating this idea (Katyal and Goldin, 2021b). We recruited long-term Tantric Yoga practitioners and matched controls who reported their perception on a binocular rivalry stimulus before and after a period of meditation while measuring EEG. During binocular rivalry, separate images are presented to the two eyes, and individuals typically perceive only one eye’s image at a time alternately with the other image every few seconds. We found that following meditation (that involved a combination of reducing *citta* and *ahamtattva*), the long-term meditators had prolonged periods of perceiving a mixture of two eye’s stimuli. Moreover, the change in mixed percept durations following meditation correlated negatively with gamma-band phase-synchronization between parietal and occipital sites, suggesting a mechanism based in decoupling of lower-order sensory regions from higher-order regions where perceptual interpretations supposedly take place (Kleinschmidt et al., 2012). Follow-up studies could combine refined qualitative methods to understand how meditator’s are phenomenologically approaching the mental object (here the rivalry stimulus) combined with neural and behavioral investigation to further understand mechanisms of reduced object and mental activity aspects of consciousness.

Reduction in object- and mental-action-related aspects of consciousness raise a critical question with regards to theories of consciousness. Is there a residual aspect of consciousness beyond these two aspects? Despite a plethora of popular consciousness theories (Graziano and Webb, 2015; Dehaene et al., 2017; Brown et al., 2019), none of them explicitly discuss such an aspect of consciousness. As mentioned above, such an idea is however consistently being discussed in recent theoretical work inspired by meditation traditions (Dunne et al., 2019; Srinivasan, 2020; Josipovic, 2021). For example, Dunne et al. (2019) use the Buddhist Mahamudra literature to propose the idea of a “non-propositional meta-awareness,” which they claim is “the aspect of consciousness that persists even after explicit focus on an object is dropped” (Dunne et al., 2019). They differentiate this aspect of consciousness from the kind of meta-awareness typically studied in psychology, which is propositional in nature with regards to mental content and is active only intermittently (e.g., a meta-awareness like: “I am [/am not] reading right now”). Conversely, non-propositional meta-awareness is a sustained phenomenal quality that runs in the background evaluating *how* one attends to the world. More recently, Metzinger (2020) has proposed that this kind of a background awareness (what he calls a “minimal phenomenal experience”) may be a Bayesian representation of basic bodily processes involved in tonic alertness. Josipovic (2021), on the other hand, proposes such an awareness to be non-representational in nature. An alternative way of conceiving reports ‘minimal phenomenal’ experiences (Shear and Jevning, 1999; Gamma and Metzinger, 2021) based on the present framework could be that they lie along a spectrum of the amount of minimal representational content (such as tonic alertness) present in consciousness combined with a non-representational object-absent aspect of consciousness. And, when the content is fully reduced, the result is a purely non-representational conscious state akin to *Savikalpa Samadhi*. Testing such a framework, while challenging, is not impossible, would require combining advances in first- and third-person approaches. For example, recent promising work on decoding mental representations using neuroimaging (e.g., Gilboa and Moscovitch, 2021) could be combined with refined empirical phenomenological methods like micro-phenomenology that help participants gain access to pre-reflective aspects of experience (Petitmengin et al., 2018; Kordes et al., 2019) that a participant may have overlooked while reporting a minimal phenomenal experience (like the duality between me and my body).

Finally, in the above account, the idea of *atman* as attained during *Nirvikalpa Samadhi* is absent any experience (and is thus meta-empirical). While this implies that the possibility of *atman* is not amenable to reflective (or pre-reflective) empirical inquiry, there are still empirical questions in need of study. According to anecdotal accounts, *Nirvikalpa Samadhi* induces in the physical body a state similar to *rigor mortis* (or postmortem rigidity), the third stage of death characterized by a stiffening of muscles (e.g., Pranavatmakananda, 2017, Chapter 14, Para 3; see https://osf.io/c4uht/ for a video document; Devashish, 2012). However, unlike postmortem where this state onsets after ∼4 h, it onsets soon after samadhi, and unlike postmortem the meditator “returns to life” after the *samadhi* ends. Such a claim warrants empirical observation, and if proven would raise the philosophical questions about possibility of a “transcendental” state beyond conscious experience.

It should also be noted that, in Tantric Yoga, the experience of pure consciousness (*mahattattva*) is considered different from pure consciousness itself (*atman*) in that the latter is devoid of experience. In the context of current literature on meditation-induced non-dual conscious states (e.g., Josipovic, 2021), this may be a feature unique to this meditation tradition. However, it is possible that other traditions also have similar concepts [e.g., the final state of emptiness (*sunyata*) in some Buddhist schools or the final state of *fanaa* in Sufism may also be one empty of experience]. Future cross-traditional work where academic experts from many different traditions collaborate with experts in philosophy of mind (e.g., Siderits et al., 2011), could help arrive at the generalizability of the structures of consciousness elaborated in the present framework more broadly beyond this tradition.

In summary, first-person inquiry of advanced Tantric Yoga meditation states may offer key insights into consciousness and its components. At the same time, Tantric Yoga practices may offer a unique method for a systematic manipulation of consciousness that can aid its empirical study in ways that have not yet been amenable to inquiry in cognitive science.

## Significance statement

Consciousness comprises a subject-object structure involving a mental action or attitude starting from the “subjective pole” upon an object of experience. “Objectless” meditation states are proposed as means to study subjective aspects of consciousness where the object of consciousness has been experientially reduced. Much of this work has been done using Buddhist meditation traditions. Another ancient Indian tradition of meditation, namely Tantric Yoga, also involves a rigorous manipulation of the subject-object structure of consciousness. Currently little is understood about how precise Tantric Yoga meditation practices could aid the study of subjective aspects of consciousness. In this paper, I expound on philosophical insight and practical procedures from a living Tantric Yoga tradition of Ananda Marga in relation to how they inform our understanding of consciousness. Specifically, Ananda Marga’s preceptor, Prabhat Ranjan Sarkar, offers philosophical developments to the ancient Indian philosophical system of Samkhya, in a way that aids contemporary understanding of consciousness in its subject-object structure. Tantric practices taught by Sarkar are then developed in this framework to allow a meditator to gradually reduce from experience the object of consciousness followed by different aspects of subjectivity. The presented work is discussed in light of existing literature on meditation and consciousness research.

## Author contributions

The author confirms being the sole contributor of this work and has approved it for publication.

## Footnotes

1 Sometimes referred to as the “Tantric tradition of Hinduism” (Gray, 2016). I refrain from using the term “Hinduism” because of its colonial nature (Pennington, 2005; Korom, 2014). Also, widely different practices come under Hindu tantra, including ones with the objective of gaining “occult powers” that I exclude from my analysis.

2 In the sense that *Prakriti* initiates both physical and mental activity upon *Purusha*, *Samkhya* is a phenomenologically derived theory of both mind/consciousness, and cosmology. One can envision that the development of such a philosophy may have had parallels to contemporary debates in consciousness studies around the “hard problem of consciousness” (Chalmers, 1995), i.e, how does the qualitative or experiential aspect of consciousness relate to something material like the brain. Indeed, one of solutions offered for resolving the hard problem is through panpsychic or cosmopsychic theories of consciousness (Brüntrup and Jaskolla, 2017; Goff et al., 2021), paralleling Yogic and Vedantic notions of consciousness (Vaidya and Bilimoria, 2015).

3 P. R. Sarkar was a prominent Indian philosopher and socio-political reformer of the 20th century (Acosta, 2010; Inayatullah, 2019). His 250 + published works (written down by his disciples based on his narrations) are divided into books on (1) philosophy and spiritual practices, and (2) topics including linguistics, socioeconomics, poetry and literature, history, agricultural practices, etc. Of these the former has been penned under the name Shrii Shrii Anandamurti while the latter under the name P. R. Sarkar.

4 Note that the way I use the word experiential reduction in this paper is different from Husserl’s phenomenological reduction. I use experiential reduction as eliminating a certain phenomenal quality from consciousness, which according to some (Bitbol, 2019) is more akin to the idea of Husserl’s epoché, or suspension of the “natural attitude.” Husserlian phenomenological reduction is then said to be the process of learning something about consciousness from the epoché, for which I use the word “deduction” here.

5 There are two key differences between the praxis-based *Tantric* adaptation of *Samkhya* philosophy and the original analytically derived *Samkhya* system. First, in the original *samkhya*, *Prakriti* is the dominant force (*pradhana*) over *Purusha*, while *Purusha* is a passive witness to *Prakriti*’s activity. Tantric adaptations, as well as Advaita Vedanta adaptations (Sharma, 2000), on the other hand assume the predominance of *Purusha* over *Prakriti*, i.e., it is the primordial “will” of *Purusha* over *Prakriti* that allows the activation of *Purusha* by *Prakriti*. A second and related difference is that while the original *Samkhya* philosophy is dualistic with *Purusha* and *Prakriti* being separate entities, Tantric and Vedantic *Samkhya* adaptations are non-dualistic. There are slight differences in Tantric and Vedantic interpretations of non-dualism, in that the former assumes a non-dual Cosmic entity *Brahma* that is a composite of *Purusha* and *Prakriti* (*Brahma* being likened to a page with its two sides being *Purusha* and *Prakriti*), whereas the later proposes a non-dual entity *Brahma* that is more equivalent to *Purusha* with *Prakriti* being casted as an “illusion.” Moreover, the idea of primordial will makes the non-dual adaptations theistic in nature.

6 This third definition also differs from Patanjali’s definition in that it has a sense of permanent cessation of mental activity akin to the Indian soteriological concept of *moksha* or *nirvana*, unlike control of mental fluctuations, which may be temporary.

7 Sarkar’s concept of *citta* can exist in various levels of “subtlety” from crude (a physical sensations) to subtle (a thought or memory) to even subtler (e.g., an intuition before it becomes a concrete thought) (Sarkar, 1993). In other words, *citta* is the phenomenological form taken by mind irrespective of whether that form is concrete or abstract.

8 Note that Sarkar appropriates terms like *citta*, *aham*, and *buddhi* (he uses *buddhi* and *mahat* as synonyms) from the classical *Samkhya* literature but assigns slightly different meanings to them.

9 In the *Ananda Marga*, long-term practice revolves around the motto “Self-realization and service to all” (*Atmamoksartham jagathitaya ca*), a phrase originally attributed to the ancient Indian text, the Rig Veda (Singh, 2005).

10 Similar to the Buddhist view, experiential dualities are considered mental “imperfections” or “bondages.” The soteriological goal of a meditator (*sadhaka*) is to be rendered free from these bondages. In Buddhism, the dualities are typically linked to the idea of *dukkha* or suffering that all beings naturally want to eliminate. In Tantra, a similar idea is framed from a positive perspective, in that consciousness in its unbound form (*purusha*) is by nature *ananda*, and the objective of “returning to” *ananda* is an inherent drive in evolution of the body and mind leading up to humans where this drive is expressed through existential curiosity (Anandamurti, 1970; Sarkar, 1993).

11 There are also more advanced lessons a meditator can learn after several decades of practice called *Vishesha Yoga* but I leave them out for the purpose of this paper.

12 Like many esoteric traditions of spiritual practice, precise *Ananda Marga* practices are imparted to the practitioner secretly. My discussion of these practices is thus based on what Sarkar has revealed publicly through his various published books combined with inputs from senior Ananda Marga practitioners (40+ or 50+ years of meditation experience) and my own 12+ years of experience.

13 Extreme dissociation is considered psychopathological. Thus, these practices are taught by ordained teachers who try to first ascertain that they would not have an adverse impact on the mental health of the meditator. Otherwise, a simpler version of the practice is taught not involving steps of *pratyahara*. According to Sarkar, a healthy social environment of meditators (or *satsaunga*) is considered essential to long-term progress in this kind of practice, which includes community support for alleviating adverse consequences of meditation. Supplementary practices that include awareness of one’s body (e.g., *asanas*) and with society (e.g., service) are considered to provide a critical balance for the mind during meditation.

14 Though individuals are assigned different *mantras* for meditation, their meaning shares the common idea of, *merging the feeling of “small I” into a “big I”* (i.e., a sense of being an individual consciousness merging into a conscious state where a separate sense of individuality does not exist).

15 The second lesson of the Ananda *Marga* meditation system is proposed to help keep the sense of individuality arising from one’s everyday behavior within bounds. This involves meditators undertaking “Cosmic ideation” (or *Brahmabhava*) within their minds before engaging in everyday actions (particularly, emotionally challenging ones). Cosmic ideation amounts to remembering that a larger transcendental entity (at the scale of the Universe) is doing the actions through them (Anandamurti, 1968). Such a lesson is supplemented by moral conduct rules (*yama* and *niyama*) to ensure that the meditators’ actions are benevolent in nature (i.e., a meditator does not engage in socially or ecologically harmful behavior while undertaking Cosmic ideation).

16 The intermediate four are considered “helper” lessons to first and sixth lessons.

17 Because this consciousness is experienced through a physical structure, it is considered inherent to matter with its ontological basis being the Yogic concept of a non-dual absolute witnessing consciousness (known as, *Parama Purusha* or *Paramatman*) characterized by *Ananda* (or bliss beyond experience). The idea of *Paramatman* is equivalent to the Yogic concept of God. The postulation of such a consciousness is often presented as an ontological argument (Oppy, 2021) – that the essence whose attribute is consciousness (as we experience it) is a property of the Universal source.
